# Correlation and responsiveness of global health, upper extremity-specific, and shoulder-specific functional outcome measures following reverse total shoulder arthroplasty for proximal humerus fracture

**DOI:** 10.1186/s12891-021-04450-y

**Published:** 2021-06-23

**Authors:** James Barger, Dafang Zhang, Derek S. Stenquist, Peter Ostergaard, Matthew Hall, George S. M. Dyer, Brandon E. Earp, Arvind von Keudell

**Affiliations:** 1 Department of Orthopaedic Surgery, Brigham and Women’s Hospital, 55 Fruit Street, MA 02115 Boston, USA; 2 Harvard Medical School, 25 Shattuck St, 02115 Boston, MA USA

**Keywords:** Proximal humerus fracture, Reverse total shoulder arthroplasty, Functional outcome, Correlation, Responsiveness, Floor effect, Ceiling effect

## Abstract

**Purpose:**

Reverse total shoulder arthroplasty (rTSA) is effective and increasingly utilized for the management of proximal humerus fracture (PHF). However, the optimal patient-reported outcome metrics (PROMs) for the evaluation of patient outcomes after this surgery are unclear. We investigated the correlation among global, upper extremity-specific, and shoulder-specific PROMs in patients undergoing rTSA for PHF as well as the responsiveness of these PROMs as assessed by floor and ceiling effects. We hypothesized that patients’ post-operative outcome would be best reflected by a combination of these metrics.

**Methods:**

Thirty patients with a history of rTSA for ipsilateral PHF filled out the following outcomes questionnaires at a minimum of 3 years post-op: EQ-5D, EQ-5D VAS, PROMIS physical function, DASH, SSV, SPADI, and ASES. Correlation between metrics was assessed using the Spearman correlation coefficient. Responsiveness was assessed by comparing the proportion of patients reaching floor or ceiling values using McNemar’s test.

**Results:**

Global health metrics (EQ-5D and PROMIS physical function) were strongly correlated with the upper extremity-specific metric (DASH). Shoulder-specific outcomes (SPADI, ASES, and ASES) were moderately correlated with both the global metrics and DASH. There was no significant difference between PROMs with regards to floor and ceiling effects.

**Conclusions:**

The DASH score has been shown to be valid and responsive for shoulder interventions, and our data demonstrate that it correlates strongly with overall quality of life. Shoulder-specific metrics are valid and responsive for shoulder interventions but correlate less with global quality of life. An optimal PROM strategy in rTSA for PHF might involve both DASH and a shoulder-specific score. Based on our assessment of floor and ceiling effects, none of these metrics should be excluded for poor responsiveness.

## Introduction

Fractures of the proximal humerus account for nearly 6 % of fractures in the adult population and the frequency of this injury is expected to increase with aging of the US population [1]. Non-operative management is indicated in the majority of cases, [2, 3] but surgery may be considered for displaced, unstable 3- and 4-part fractures, head-split fractures, and fracture-dislocations [4]. While the optimal mode of surgical management of proximal humerus fractures (PHFs) continues to be debated, [4] the use of reverse total shoulder arthroplasty (rTSA) for the surgical treatment of complex PHFs is increasing [5–7]. Numerous studies have demonstrated the effectiveness of rTSA and better outcomes for rTSA than hemiarthroplasty, though no randomized study has demonstrated superior outcomes from rTSA relative to non-operative management [7–13]. Prior studies on rTSA for fracture have reported patient outcomes using a variety of global health, upper extremity-specific, and shoulder-specific functional scores [4, 14].

Patient-reported outcome metrics (PROMs) attempt to capture the biopsychosocial impact of disease on patients’ lives [15] and are becoming more important than ever in orthopaedic surgery with the shift in healthcare from fee-for-service to value-based care [16]. They allow the patient’s subjective experience of their injury and its treatment to be formally assessed, in addition to traditional objective and clinician-reported measures, which tend to underestimate patient symptoms and functional limitations [17, 18]. Health-related quality of life (HRQoL) is defined as how a person’s health affects his or her ability to carry out normal social and physical activities [19]. Surgery for a PHF is intended to restore HRQoL and reduce disability, but the relationship between overall HRQoL and extremity-specific function for this condition is not known. In addition, PROMIS scores are increasingly used to evaluate patient functional outcomes but they have not been widely studied for upper extremity conditions [16]. Understanding associations between global health, upper extremity-specific, and shoulder-specific outcomes after rTSA for fracture will help surgeons to better measure the results of this treatment on patients’ lives.

The objectives of this study were (1) to assess correlations among global health, upper extremity-specific, and shoulder-specific functional outcome measures for rTSA performed for proximal humerus fractures at minimum 3-year follow-up, and (2) to compare the responsiveness of global health, upper extremity-specific, and shoulder-specific functional outcome measures by assessing floor and ceiling effects.

## Methods

### Study design and patient selection

The study was designed following the COnsensus-based Standards for the selection of health status Measurement INstruments (COSMIN) guidelines for studies on measurement properties and specifically those guidelines regarding construct validity and responsiveness [20]. The study meets COSMIN criteria for these psychometric parameters, though a larger patient population (> 50) would have been preferred. Other psychometric parameters of the PROMs included in the study, including internal consistency and content, structural, and cross-cultural validity were outside the scope of this study but have been reported by previous investigators.

 After obtaining approval from the Institutional Review Board, a retrospective analysis was performed of all patients who underwent rTSA for proximal humerus fractures at two American College of Surgeons Level I trauma centers from January 2003 to June 2017. Using our institutions’ Research Patient Data Registry (RPDR), patients were identified using the International Classification of Disease (ICD-9 and ICD-10) codes for the diagnosis of proximal humerus fracture and Current Procedural Terminology (CPT) code for shoulder arthroplasty. Five hundred seventy-six patients were identified from the initial screen. Inclusion criteria were patients 18 years of age or older who underwent rTSA for a proximal humerus fracture between January 1, 2003 and August 1, 2018 and had a minimum of 3 years postoperative follow-up. Exclusion criteria included diagnoses other than proximal humerus fracture, procedures other than rTSA, surgeries performed at an outside institution, and patients without documented postoperative follow-up.

We excluded 264 patients for a diagnosis other than proximal humerus fracture, 74 patients who did not undergo rTSA, and 14 patients who had rTSA for fracture performed at an outside institution. The remaining 224 patients were contacted by telephone at a minimum of 3 years following rTSA for our outcome measures; 71 patients were further excluded for inadequate follow-up, of which 26 were deceased and 55 were unavailable. Furthermore, 113 patients were excluded for an incomplete set of outcome measures at a minimum of 3 years following rTSA, 112 because they were not sent all of the functional outcomes surveys due to their inclusion in different research protocols, and 1 who was contacted for all scores but returned EQ-5D, PROMIS, and DASH but not ASES, SPADI, or SSV. The final cohort included 30 patients (30 shoulders) with proximal humerus fractures treated with rTSA with a complete set of functional outcome measures at least 3 years of follow-up.

### Outcome measures

The outcome measures in this study included global health outcome measures, an upper extremity-specific outcome measure, and shoulder-specific outcome measures. Global health outcome measures included the Patient-Reported Outcomes Measurement Information System (PROMIS) Global Health Physical Score Short Form 10b, EuroQol Five-Dimension Score (EQ-5D), and EuroQol Visual Analogue Scale (EQ-VAS). The upper extremity-specific outcome measure was the Disabilities of the Arm, Shoulder and Hand (DASH) score. Shoulder-specific outcome measures included the Shoulder Subjective Value (SSV), American Shoulder and Elbow Surgeons (ASES), and Shoulder Pain and Disability Index (SPADI). All outcome measures were collected through review of the medical records and telephone contact at a minimum of 3 years following rTSA.

The PROMIS Physical Function instrument is a 10-item questionnaire that evaluates physical, mental, and social aspects of health, and all items are evaluated on a 5-point Likert scale [10]. The questionnaire has Physical and Mental Health subscales. The Physical Health subscale score, which was utilized in our study, ranges from 0 to 100 with a mean score of 50 representing the norm for the US general population and a standard deviation of 10. Higher scores indicate better health [16, 21].

The EQ-5D is a health-related quality of life measure with 5 domains: mobility, self-care, usual activities, pain/discomfort, and anxiety/depression [21] There are three options for each response. A score of 1.0 represents perfect health while scores less than zero indicate health status worse than death [21]. The EQ-VAS is a separate quantitative self-assessment of health which has patients evaluate their own health on a visual analogue scale from 0 to 100 where 0 is worst and 100 is best [21].

The DASH score is a 30-item upper-extremity-specific outcome measure which is intended to measure shoulder, elbow, hand, and wrist function in one combined metric [22]. It does not discriminate between the affected and non-affected upper extremity. The DASH assesses multiple domains including physical function, symptoms, and social/psychological function [13]. It has been shown to correlate with shoulder-specific measures, [23]. The DASH is scored from 0 to 100, with lower scores representing better function. Normative values are 22 in females and 13 in males aged 70 to 79 [24]. The minimum clinically important difference (MCID) for shoulder conditions is approximately 10 [this MCID value, and those reported for other metrics below, is not intended to suggest that the MCID in our patient population is identical, but merely to give a sense of the scale on which clinically important differences have been previously reported to occur for other shoulder pathologies] [22].

The SSV is an entirely subjective shoulder-specific measure where the patient is asked to rate their shoulder on a scale from 0 to 100 with some version of the following question: “What is the overall percent value of your shoulder if a completely normal shoulder represents 100 %?” [25] The SSV is considered an adjunct to more complicated shoulder-specific scores and has been shown to be an easily administered, responsive, and valid measure of shoulder function [25].

The ASES is a shoulder-specific score which consists of 10 functional questions and a pain VAS score [22]. The total score ranges from 0 to 100 (higher is better) and is weighted 50 % for function and 50 % for pain. The MCID is considered to be 6.4 for various shoulder pathologies [22].

The SPADI is a self-assessment of symptoms and function of the shoulder designed for any disorder of the shoulder joint [26]. It consists of 13 items (5 for pain symptoms and 8 for disability) which are scored on a 0 to 100 point visual response scale. The domain scores are combined to produce a score from 0 to 100 where 0 is best and 100 is worst [27]. The MCID for the SPADI has been reported to be between 8 and 13.2 [27].

Patient demographics were collected through our institutional RPDR and review of the electronic medical records, including age, sex, hand dominance, body mass index (BMI), Charlson Comorbidity Index (CCI), diabetes mellitus, depression, osteoporosis, smoking status at presentation, and employment status. Injury-related variables included fracture type, presence of ipsilateral upper extremity fracture, open fracture, and associated nerve injury. Treatment-related variables included initial treatment (acute rTSA versus nonoperative treatment), tuberosity repair, complications, and reoperation. The operative techniques and rehabilitation protocols were at the discretion of the treating surgeon.

### Statistical analysis

Descriptive statistics were calculated for the study cohort. Measurement of correlations between outcome measures were performed using the Spearman correlation coefficient. To study floor and ceiling effects, the proportion of patients reporting the minimum and maximum scores of each outcome measure, respectively, were determined. Paired comparisons of the proportion of patients at the floor and ceiling of outcome measures were performed using McNemar’s test. The standard significance criterion of α = 0.05 was employed. A convenience sample was used.

## Results

### Cohort demographics

Our study cohort comprised 30 patients treated with rTSA following proximal humerus fracture. The average age of our cohort was 70.2 years at the time of surgery, and 87 % were female. Ten (33 %) of patients had diabetes mellitus, 6 (20 %) had depression, 6 (20 %) had osteoporosis, and 1 (3 %) smoked at the time of surgery. The dominant upper extremity was affected in 18 (60 %) of patients, and 6 (20 %) of patients were actively employed. The tuberosity was repaired during rTSA in 20 (67 %) of patients (Table 1).

Twenty-three out of 30 (77 %) patients were treated with rTSA acutely, and 7 (23 %) were treated with rTSA after failed initial nonoperative treatment. Complications occurred in four patients and included instability, infection, heterotopic ossification, and periprosthetic fracture. Two patients (7 %) underwent reoperation, which included component revision for instability and irrigation and debridement with liner exchange for infection. The median clinical follow-up after rTSA was 17.5 months (IQR 10.5 to 30.1 months). The median time to telephone assessment of functional outcome measures was 5.2 years (IQR 4.3 to 6.7 years, minimum 59 days).

**Table 1:** Baseline characteristics of rTSA for fracture patients (*n* = 30)

	**Mean (Standard Deviation)**
**Age**	70.2 (6.7)
**BMI**	30.1 (5.1)
	**Median ** **(Interquartile Range)**
**CCI**	3 (2–5)
	**n (%)**
**Male sex**	4 (13)
**Diabetes mellitus**	10 (33)
**Depression**	6 (20)
**Osteoporosis**	6 (20)
**Current smoker**	1 (3)
**Employed**	6 (20)
**Dominant upper extremity injury**	18 (60)
**Ipsilateral upper extremity injury**	1 (3)
**Nerve injury**	1 (3)
**Cemented humeral stem**	14 (47)
**Tuberosity repaired**	20 (67)

### Correlation between outcome measures

Global health, upper extremity-specific outcome measure, and shoulder-specific outcome measures were collected for the cohort at minimum 3-year follow-up after rTSA for fracture (Table 2).

**Table 2:** Functional outcome scores of rTSA for fracture patients (*n* = 30)

	Mean (Standard Deviation)
**PROMIS**	43.7 (9.9)
**EQ-5D**	0.77 (0.13)
**EQ-VAS**	76.5 (13.6)
**DASH**	25.2 (16.7)
**SSV**	78.4 (21.5)
**SPADI**	26.4 (18.8)
**ASES**	73.6 (20.7)

**Fig. 1:**
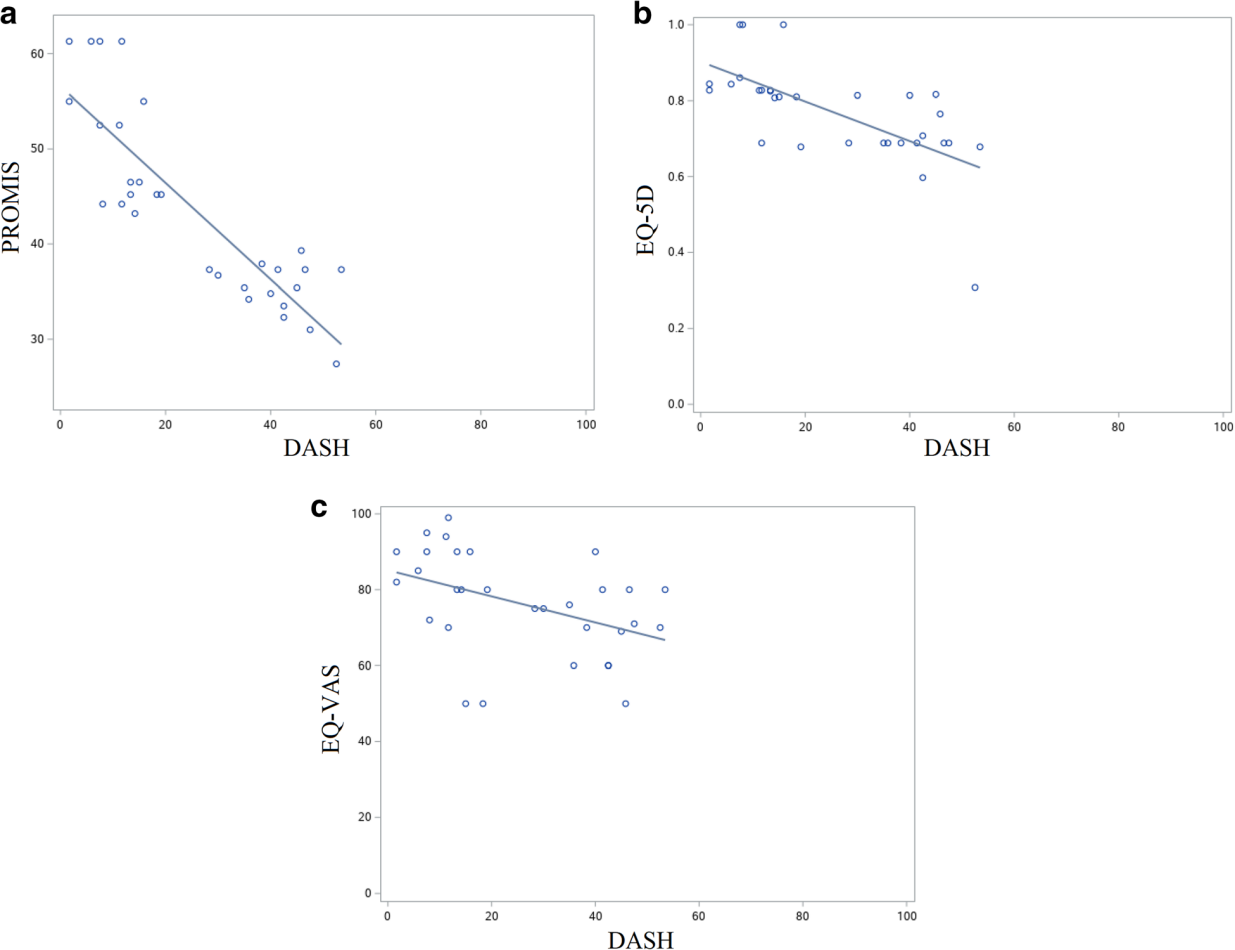
Scatter plots with linear regression lines showing correlation between global health and upper extremity-specific outcome measures

Global health and upper extremity-specific outcome measures were compared (Fig. 1). PROMIS and DASH (*ρ* = -0.85, *p* < 0.0001) and EQ-5D and DASH (*ρ* = -0.75, *p* < 0.0001) were highly correlated. EQ-VAS and DASH (*ρ* = -0. 45, *p* = 0.002) were moderately correlated.

**Fig. 2:**
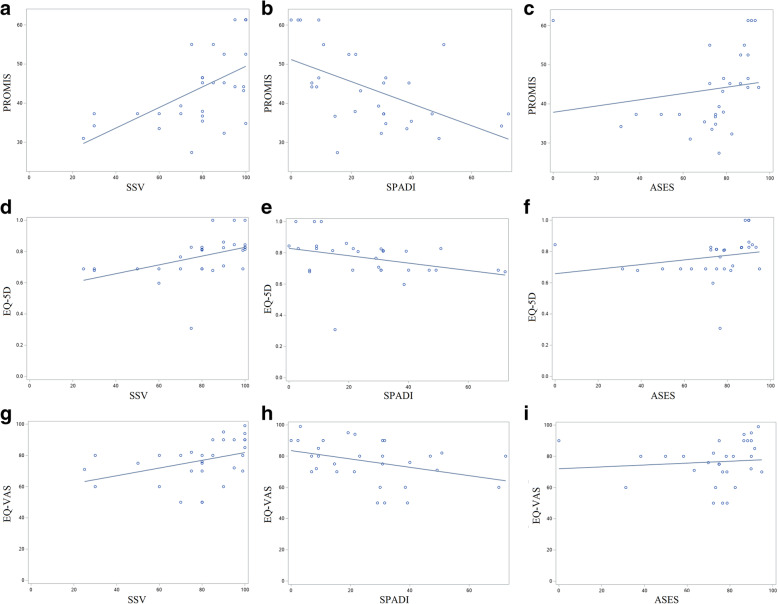
Scatter plots with linear regression lines showing correlation between global health and shoulder-specific outcome measures

Global health and shoulder-specific outcome measures were compared (Fig. 2). PROMIS and SSV (*ρ* = 0.58, *p* = 0.0007), PROMIS and SPADI (*ρ* = -0.55, *p* = 0.002), and PROMIS and ASES (*ρ* = 0.51, *p* = 0.004) were moderately correlated. EQ-5D and SSV (*ρ* = 0.63, *p* = 0.0002), EQ-5D and SPADI (*ρ* = -0.47, *p* = 0.008), and EQ-5D and ASES (*ρ* = 0.48, *p* = 0.007) were moderately correlated. EQ-VAS and SSV (*ρ* = 0.55, *p* = 0.002) were moderately correlated, but EQ-VAS and SPADI (*ρ* = -0.35, *p* = 0.06) and EQ-VAS and ASES (*ρ* = 0.29, *p* = 0.1) were not significantly correlated.

**Fig. 3:**
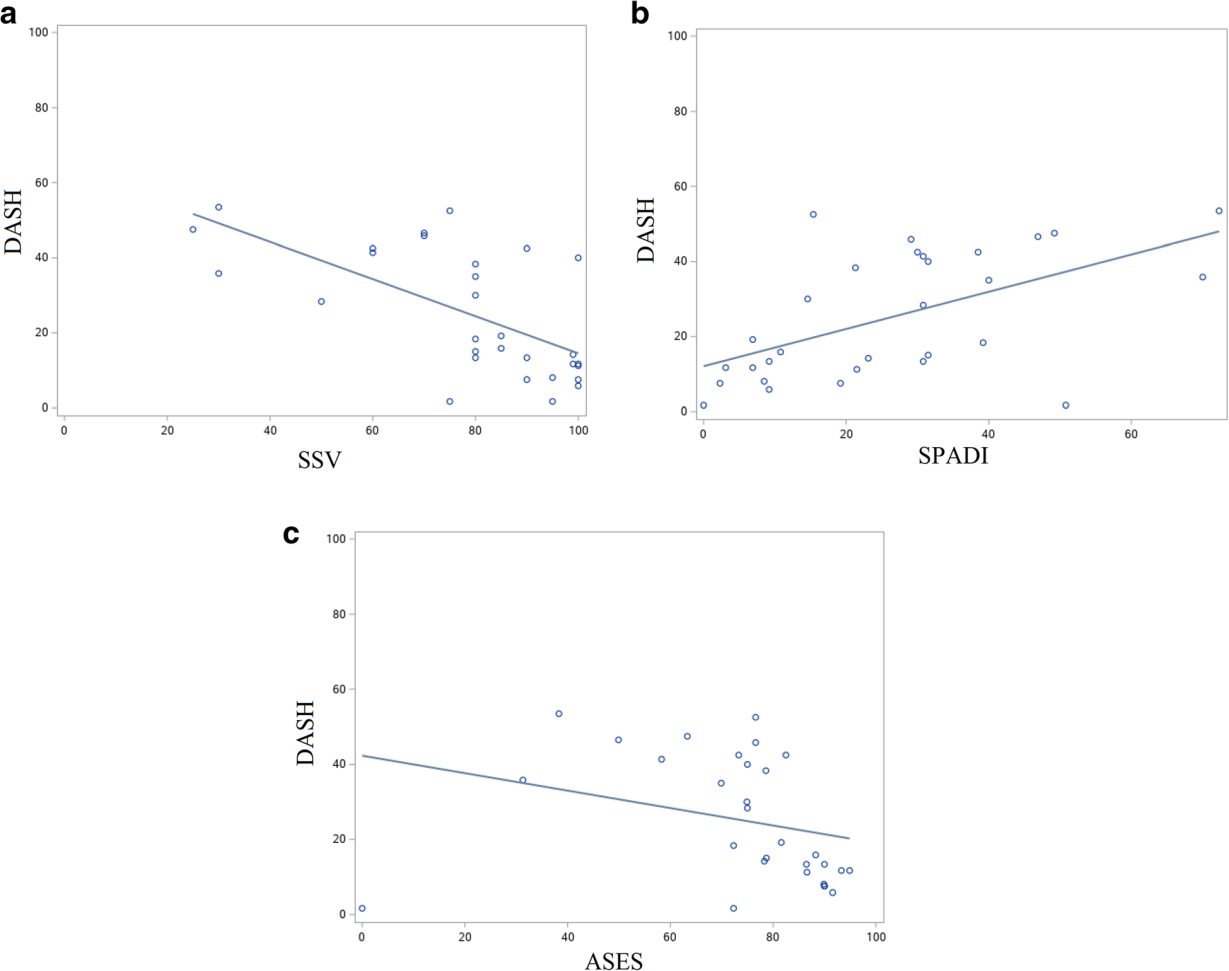
Scatter plots with linear regression lines showing correlation between upper extremity-specific and shoulder-specific outcome measures

Upper extremity-specific and shoulder-specific outcome measures were compared (Fig. 3). DASH and SSV (*ρ* = -0.67, *p* < 0.0001), DASH and SPADI (*ρ* = 0.55, *p* = 0.002), and DASH and ASES (*ρ* = -0.52, *p* = 0.003) were moderately correlated.

Among global health outcome measures, PROMIS and EQ-5D (*ρ* = 0.73, *p* < 0.0001) were strongly correlated, whereas PROMIS and EQ-VAS (*ρ* = 0.59, *p* = 0.0005) and EQ-5D and EQ-VAS (*ρ* = 0.54, *p* = 0.002) were moderately correlated. Among shoulder-specific outcome measures, SSV and SPADI (*ρ* = -0.67, *p* < 0.0001), SPADI and ASES (*ρ* = -0.68, *p* < 0.0001), and SSV and ASES (*ρ* = 0.69, *p* < 0.0001) were moderately correlated.

### Responsiveness of outcome measures

Five patients reported the maximum SSV score, 3 patients reported the maximum EQ-5D score, and 1 patient reported the maximum SPADI score. No other functional outcome measures exhibited a ceiling effect.

One patient reported the minimum ASES score. No other functional outcome measure exhibited a floor effect.

McNemar’s tests did not demonstrate a significant difference between the proportion of patients at the ceilings of EQ-5D and SSV (*p* = 0.4) and EQ-5D and SPADI (*p* = 0.3).

## Discussion

There are numerous PROMs available for the assessment of HRQoL and function following shoulder surgery. In selecting which measures to collect from patients, it is important to understand which aspects of the patient’s experience are reflected by each score and to avoid unnecessary redundancy [28]. Rates of survey completion by patients has been shown to correlate negatively with survey length, suggesting that it is important to select outcome measures judiciously [28]. PROMs collected in previous studies on rTSA for fracture have varied widely, so our study sought to investigate the contribution of and relationship between several commonly used metrics [4, 14].

The correlation between global outcome measures (EQ-5D and PROMIS physical function) and DASH was stronger than that between global outcomes and shoulder-specific measures (ASES, SPADI, and SSV). This likely reflects the fact that the DASH is a comprehensive evaluation of upper-extremity function that captures the effect of an upper-extremity intervention (rTSA for fracture, in this case) on a patient’s overall quality of life. As such, it can be interpreted as a proxy for the effect of an upper extremity intervention on global HRQoL, while simultaneously focusing on the upper extremity and thereby being more sensitive to treatment effect than truly global metrics like the EQ-5D. This hypothesized increased sensitivity is borne out by the fact that the data in this study was used in a separate project, not yet published, to compare outcomes between those undergoing rTSA for fracture acutely versus in a delayed manner for mal- or non-union. While there was a significant difference in DASH scores; EQ-5D and PROMIS did not detect this group difference.

Shoulder-specific outcomes scores (ASES, SPADI, and SSV) correlated only moderately with DASH scores and global metrics. These scores focus explicitly on shoulder function, and while they may be responsive to shoulder interventions, are less reflective of overall quality of life. These findings suggest that the optimal selection of outcomes scores in evaluating rTSA for fracture may include DASH score, which offers upper-extremity focus while correlating well with global HRQoL, plus a shoulder-specific score to optimize responsiveness to this shoulder intervention. Our findings also support the use of the PROMIS instrument as an easy-to-use alternative to the well-established but lengthier DASH questionnaire. Since we found significant correlation among shoulder-specific outcome measures (ASES, SPADI, and SSV), we recommend collecting only one shoulder-specific score to avoid redundancy. Our study supports the construct validity of the Subjective Shoulder Score as an easily obtained metric that reflects patient quality of life as it relates to the shoulder. The SSV correlated more closely (though still just moderately) with DASH, PROMIS, and EQ-5D than ASES or SPADI and involves only one simple question. Therefore, for researchers who feel collecting the DASH metric in addition to a shoulder-specific score may add undue burden, the SSV might be a reasonable alternative.

 While collecting both DASH and a shoulder-specific metric seems to be a good combination following rTSA for fracture, this raises the question of whether two PROMs may introduce survey fatigue, particularly as the DASH contains 30 questions and is therefore one of the longer survey metrics available. A meta-analysis of survey burden in PROMs by Rolstad et al. demonstrated that increased survey length correlates with decreased response rate. However, there was no particular survey length at which responses dropped off and the willingness of patients to complete longer surveys varied between conditions [28]. As such, there is no clear answer to how much is “too much.” Atkinson et al. found minimal patient-reported survey burden in surgical oncology patients completing the 30-question EORTC QLQ-C30 metric, whether or not it was coupled with a 30-minute interview on their experiences of illness [29]. This was despite the fact that many subjects noted redundancy and suggests that patients are generally willing to spend considerable time reporting on conditions which have greatly affected their lives. As such, we feel that collecting two outcome metrics is unlikely to decrease survey response, but cannot conclude this definitively. This decision ultimately resides with clinician-researchers. In our study, of the 31 patients who were contacted for all six PROMs and agreed to participate, just one failed to complete them all, suggesting that the survey burden of two PROMs is unlikely to be an obstacle to data collection in patients undergoing rTSA for fracture.

In addition to investigating the correlation between outcomes scores in patients undergoing rTSA for fracture, our study sought to assess whether the responsiveness of these scores were affected by floor or ceiling effects. The floor or ceiling effect describes the circumstance in which patient outcome scores cluster at the minimum or maximum value for a given metric, thus rendering the outcome measure unable to detect differences between these patients [30]. At least one patient reported the maximum value for three of the metrics (SSV, EQ-5D, and SPADI), but for each of these metrics the number of patients at the ceiling was small, and we found no significant difference in the proportion of patients at the ceiling. It is unsurprising we did not find a ceiling effect, given that these patients did not necessarily have any pathology prior to their fracture and thus their pre-injury comparison was likely to a fairly normal shoulder. More interesting is the fact that only one patient reported a minimum value, for the ASES. These findings suggest that the responsiveness of the outcomes we measured is not affected by floor or ceiling effects in rTSA for fracture.

There are numerous additional outcomes metrics available for the study of shoulder interventions which were not included in our study. Perhaps the most widely used instrument in the proximal humerus fracture literature is the Constant-Murley score. We elected not to collect this score and feel it is not the metric of choice in studies of rTSA after fracture for multiple reasons. First, it contains a physical exam component which includes range of motion and power. This introduces the risk of bias by the examiner, may be inappropriate to combine with patient-reported domains, and is conflated by normal age-related declines in shoulder strength [31]. Second, several studies have questioned its reliability and correlation with other shoulder outcomes metrics [31]. Two other metrics which were not included in out study but which are widely used in shoulder arthroplasty registries are the Western Ontario Osteoarthritis of the Shoulder (WOOS) index (particularly used in Scandinavian registries) and the Oxford Shoulder Score (OSS) (widely used in Commonwealth registries). The WOOS is a disease-specific metric designed to be particularly responsive to interventions for shoulder osteoarthritis [32]. The Pearson correlation coefficient between the WOOS and ASES has been reported between 0.570 in the original validation study to 0.88 more recently in patients undergoing rTSA for OA, representing a moderate to excellent correlation [32, 33]. There is also strong correlation between the WOOS and DASH (*r* = 0.75) [34]. These data suggest that there is translatability between the WOOS and the metrics in our study. The OSS has been reported to have excellent correlation with the ASES (*r* = 0.91) [35] and moderate correlation with DASH (*r*=-0.059) [36], suggesting translatability of the OSS as well.

Limitations of this study include the fact that several widely utilized PROMs were not included due to concern for patient survey fatigue. We did not collect the questionnaires at multiple pre-determined time points and thus were unable to compare the proportion of patients reaching clinically important differences, which would have better allowed us to assess the responsiveness of the scores over time. Finally, the patient population was on the smaller side of the acceptable range recommended by COSMIN guidelines.

PROMs allow the patient’s subjective experience to be reflected in surgical outcomes, but there has been little consistency in the literature as to which PROMs should be used to track outcomes after rTSA for fracture. Our results suggest that use of a global health or upper extremity-specific score, such as PROMIS physical function or DASH, in conjunction with a shoulder-specific metric, such as SSV, SPADI, or ASES, may strike an optimal balance. The PROMIS physical function score and SSV are appealing instruments, because they correlate well with other relevant metrics and are more easily administered. There was no significant difference in floor or ceiling effects between any of the metrics in our study.

## Data Availability

De-identified data may be requested from the corresponding author via email.
